# Nanosecond X-ray photon correlation spectroscopy using pulse time structure of a storage-ring source

**DOI:** 10.1107/S2052252520015778

**Published:** 2021-01-01

**Authors:** Wonhyuk Jo, Fabian Westermeier, Rustam Rysov, Olaf Leupold, Florian Schulz, Steffen Tober, Verena Markmann, Michael Sprung, Allesandro Ricci, Torsten Laurus, Allahgholi Aschkan, Alexander Klyuev, Ulrich Trunk, Heinz Graafsma, Gerhard Grübel, Wojciech Roseker

**Affiliations:** a Deutsches Elektronen-Synchrotron (DESY), Notkestr. 85, 22607 Hamburg, Germany; b Institute of Physical Chemistry, University of Hamburg, Grindelallee 117, 20146 Hamburg, Germany; c The Hamburg Centre for Ultrafast Imaging, Luruper Chaussee 149, 22761 Hamburg, Germany

**Keywords:** materials science, nanoscience, SAXS, dynamical studies, time-resolved studies, X-ray photon correlation spectroscopy, adaptive gain integrating pixel detectors, storage rings, pulse structures

## Abstract

X-ray photon correlation spectroscopy (XPCS) is a routine technique to study slow dynamics in complex systems at storage-ring sources. Achieving nanosecond time resolution with the conventional XPCS technique is, however, still an experimentally challenging task requiring fast detectors and sufficient photon flux. Here, the result of a nanosecond XPCS study of fast colloidal dynamics is shown by employing an adaptive gain integrating pixel detector (AGIPD) operated at frame rates of the intrinsic pulse structure of the storage ring. Correlation functions from single-pulse speckle patterns with the shortest correlation time of 192 ns have been calculated. These studies provide an important step towards routine fast XPCS studies at storage rings.

## Abstract 2

Nanosecond XPCS study of fast colloidal dynamics is demonstrated by employing the intrinsic pulse structure of the storage ring with AGIPD. Correlation functions from single-pulse speckle patterns with the shortest correlation time of 192 ns have been measured, providing an important step towards routine ultrafast XPCS studies at storage rings.

## 1. Introduction   

X-ray photon correlation spectroscopy (XPCS) is a very powerful and well established tool to investigate slow dynamics of various complex disordered systems in condensed matter, such as colloids (Dierker *et al.*, 1995 ▸; Grübel & Zontone, 2004 ▸; Li *et al.*, 2014 ▸), polymers (Lehmkühler *et al.*, 2018 ▸; Frenzel *et al.*, 2019 ▸), capillary waves (Gutt *et al.*, 2003 ▸), metallic glasses (Ruta *et al.*, 2012 ▸; Evenson *et al.*, 2015 ▸), molecular glasses (Chushkin *et al.*, 2012 ▸), charge-density waves (Shpyrko *et al.*, 2007 ▸) and water (Perakis *et al.*, 2017 ▸, 2018 ▸). XPCS is derived from well established visible photon correlation spectroscopy (PCS) (Provencher, 1979 ▸; Dorfmüller, 1992 ▸; Cipelletti & Weitz, 1999 ▸), where a visible laser acts as a coherent source. The development of bright X-ray sources such as third-generation synchrotrons allowed the PCS technique to be extended to high-scattering vectors from typically 10^−3^ to a few 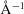. As the wavelength of the probe beam defines the spatial resolution, using X-rays as the probe is crucial to investigating atomic scale dynamics (Leitner *et al.*, 2009 ▸). XPCS is based on illuminating a disordered sample with a coherent photon beam. A grainy interference pattern, commonly called a ‘speckle’ pattern (Sutton *et al.*, 1991 ▸), is formed in the far field, where typically a 2D area detector with high spatial resolution is placed. The speckle pattern contains information on the exact spatial arrangement of the sample disorder. If the spatial arrangement changes with time, the speckle pattern will also change. The 2D detector measures a time series of speckle patterns and can reveal dynamical information about the sample encoded in the time-dependent speckle patterns.

In conventional XPCS, the temporal resolution is defined by the number of 2D X-ray speckle patterns that can be measured in time. The upper limit is given by either the detector frame rate or the X-ray pulse repetition rate. Although most of the third-generation synchrotron-radiation facilities provide MHz pulse repetition rates, commercially available 2D X-ray detector systems offer frame rates up to the kHz region. Therefore, most XPCS experiments have been performed typically on systems that show dynamics from minutes down to milliseconds (Perakis *et al.*, 2017 ▸; Dierker *et al.*, 1995 ▸; Thurn-Albrecht *et al.*, 1996 ▸; Ruta *et al.*, 2012 ▸).

During recent years the access to faster dynamics has been enabled by hard X-ray free-electron laser sources (XFELs) (Emma *et al.*, 2010 ▸; Ishikawa *et al.*, 2012 ▸; Kang *et al.*, 2017 ▸; Decking *et al.*, 2020 ▸) via split-pulse XPCS (Roseker *et al.*, 2018 ▸, 2020 ▸) and X-ray speckle visibility spectroscopy (XSVS) (Perakis *et al.*, 2018 ▸). In both techniques, the time resolution is independent of the detector frame rate. However, the split-pulse technique requires special X-ray optics and a split-and-delay device (Roseker *et al.*, 2009 ▸; Zhu *et al.*, 2017 ▸; Osaka *et al.*, 2016 ▸; Rysov *et al.*, 2019 ▸), and XSVS relies on femtosecond-precise control of the pulse length.

There have been strong improvements in X-ray detector response time and efficiency. Commercially available detectors such as the large-area medipix-based detector array (LAMBDA) or EIGER allow one to measure with 2 kHz (Pennicard *et al.*, 2012 ▸) and 20 kHz (Johnson *et al.*, 2012 ▸) frame rates, respectively. Also, the prototype VX-798 with a two-gates system that can be triggered with fine time resolution (Ross *et al.*, 2016 ▸) was demonstrated in two-pulse XPCS measurements with 120 µs temporal resolution (Dufresne *et al.*, 2016 ▸). Recently, an XPCS study was performed with 1.13 MHz frame rate at the European XFEL using AGIPD (Lehmkühler *et al.*, 2020 ▸).

Here, we demonstrate an XPCS study with 192 ns temporal resolution by employing the pulse structure of the PETRA III storage ring and AGIPD. Thanks to the MHz frame rate of the detector, we could successfully acquire scattering data from single storage-ring pulses with sufficient statistics. Our result shows the expected dynamics of the investigated system and demonstrates the successful application of AGIPD at storage-ring sources.

## 2. Experiment   

**Figure 1:**
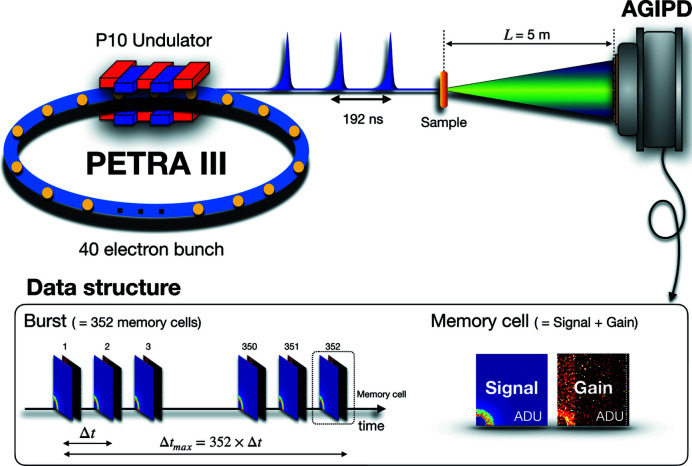
Schematics of the XPCS experiment employing the 40-bunch mode of PETRA III. X-ray pulses are delivered to the sample position with 192 ns time separation. The resulting speckle pattern from each X-ray pulse is recorded by AGIPD.

The experiment was carried out at the P10 beamline of PETRA III using 8 keV X-rays monochromated by a cryogenic cooled Si(111) crystal monochromator. The photon beam was focused to a size *d*_b_ of 2.5 × 2.5 µm (*H* × *V*) at the sample position using a 2D transfocator Be lens system (Zozulya *et al.*, 2014 ▸). X-ray pulses were delivered to the second experimental hutch EH2 with Δ*t* = 192 ns temporal separation, defined by the electron-bunch spacing in the 40-bunch mode of PETRA III (see Fig. 1 ▸).

The detector was located at a distance *L* = 5 m from the sample providing a maximum scattering wavevector *Q* of 0.14 nm^−1^. The expected speckle size was estimated according to *d*_s_ = λ*L*/*d*_b_ = 310 µm, where λ is the X-ray wavelength. Based on the speckle and the AGIPD pixel size (*d*_p_ = 200 µm), the expected speckle contrast (β) is 0.65 (see Fig. S1 of the Supporting information).

Colloidal silica particles with a radius of 180 nm were dispersed in water and placed in an 0.7 mm glass capillary. The static properties of the sample (*i.e.* polydispersity and size of the particles) were measured with a LAMBDA 750k detector, composed of 512 × 1536 pixels each having a size of 55 × 55 µm. The dynamics of the sample were investigated using AGIPD. Single application-specific integrated circuit (ASIC) (Shi *et al.*, 2010 ▸; Mezza *et al.*, 2016*a* ▸, 2016*b* ▸) hybrid assembly of AGIPD was employed in the measurements. The active area of the ASIC is 1.28 × 1.28 cm, composed of 64 × 64 pixels. The data structure is composed of bursts and memory cells (see Fig. 1 ▸). A single burst stores data in 352 memory cells with two data matrices, one for analog-signal value and one for corresponding gain information. In our experiment, X-ray pulses were generated from the PETRA III storage ring with 5.2 MHz. The minimum delay step between the memory cells was adapted to the separation between the X-ray pulses (δ_*t*_ = 192 ns).

## 3. Gain correction and background subtraction   

The in-pixel memory of the detector has a significant advantage for overcoming a hardware limitation of long readout times which strongly restricts the maximum operating frequency of a detector. In addition, the novel adaptive gain system automatically switches the gain states in each pixel according to the exposed intensity for every memory cell during the measurement. The three available gain states are stored as encoded analog-to-digital unit (ADU) values (Mezza *et al.*, 2016*a* ▸). Any variations of the scattering data signals and gain values caused by nonuniformities of the storage cells, especially during the fast-acquisition mode, have to be investigated. Therefore, the extraction of gain states and background subtraction is a crucial procedure prior to analyzing data.

### 3.1. Multi-pulse mode   

**Figure 2:**
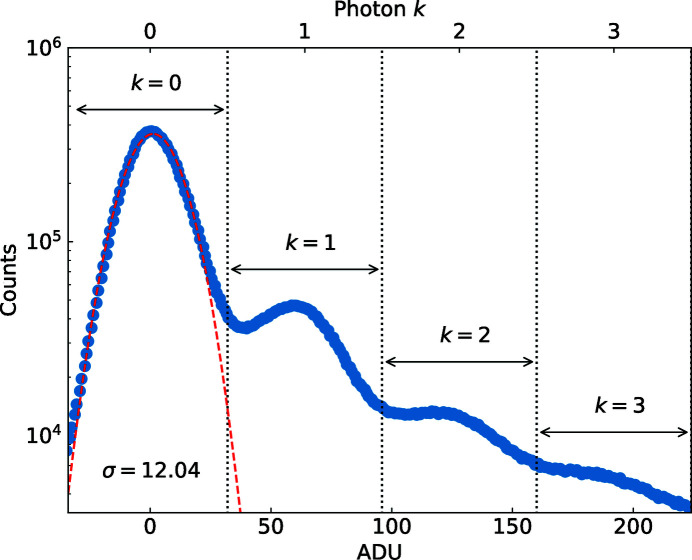
A histogram of ADUs obtained from MP mode. The vertical dashed lines show selected ranges for the conversion of ADUs into an integer number of photons. Photon peaks occur at multiples of 64 ADUs.

We employed the so-called multi-pulse (MP) mode (*i.e.* 520 X-ray pulses accumulated in one memory cell during 100 µs exposure time) to access a temporal scan range from 100 µs to 35.2 ms. In total, *N*_b_ = 11 bursts were measured. The encoded gain states of every pixel and memory cell were extracted carefully from baseline variation (Mezza *et al.*, 2019 ▸). In this case, simply histogramming the scattering data to define gain states (Mezza *et al.*, 2016*a* ▸; Allahgholi *et al.*, 2019 ▸) was not sufficient. Therefore, we used dark frames (*i.e.* operating the detector without X-ray exposure) to eliminate baseline variations. Fig. 2 ▸ shows an ADUs histogram of the MP mode after defining the gain states by taking into account the gain of dark data. The second peak of the histogram is centered at 64 ADUs and corresponds to the first photon peak. The detailed data analysis is explained in the Supporting information in Sections S2 and S3. The noise of the detector was derived from the σ value (12 ADUs) of the zero photon peak.

### 3.2. Single-pulse mode   

We were able to successfully resolve 352 single shots of X-ray speckle patterns in the fast-acquisition mode of AGIPD. The pulses were temporally separated by 192 ns, which corresponds to the time between the X-ray pulses in the 40-bunch operation mode of the storage ring. In this acquisition mode, the scattering intensity is ∼520 times lower than the MP mode owing to the exposure time of a single frame. The gain value of all pixels remains in high gain (called Gain I in the following) and therefore no gain switching occurs during the measurement time (see Section S3). Accordingly, gain switching was not taken into account for background subtraction in the single pulse (SP) acquisition mode. In order to subtract the background from the raw data, we calculated the median value 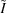 of intensity in a single pixel of a single memory cell, according to 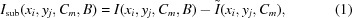
where *C* and *B* are memory cell and burst number, respectively. The *i* and *j* are pixel indices, and *m* represents the memory cell index. *N*_b_ = 2850 bursts were measured to give sufficient statistics for the median calculation.

## 4. Results   

**Figure 3:**
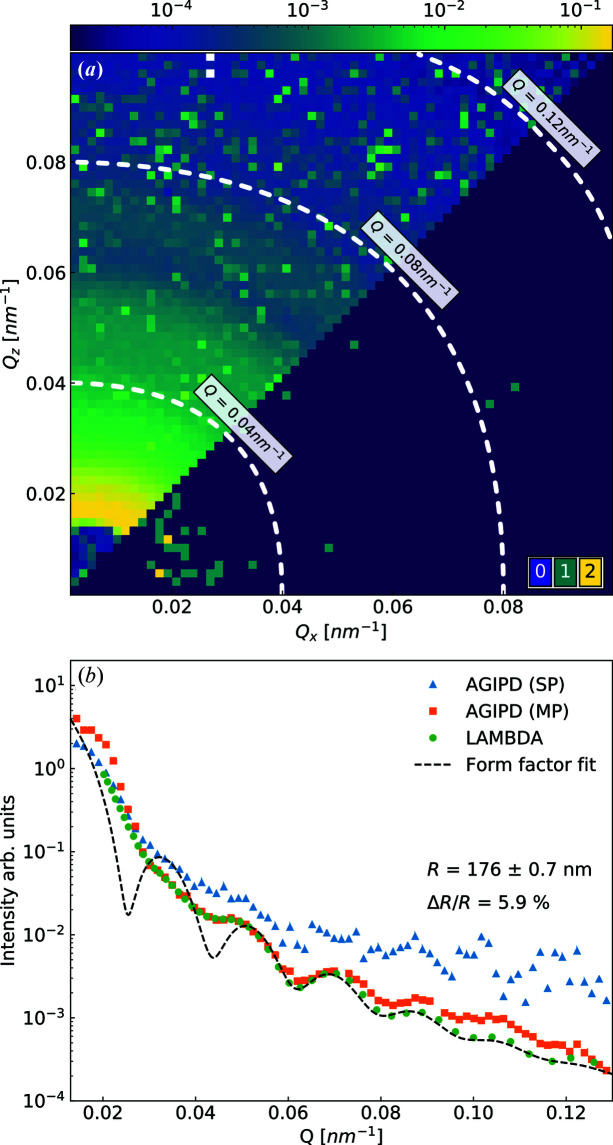
(*a*) A scattering pattern from silica particles measured with AGIPD in SP mode. A SP image is shown in the bottom-right part of the image. The upper-left part of the image shows the result of a sum of 2850 × 352 frames. The dashed lines represent the corresponding *Q* values in the text boxes. (*b*) Azimuthally averaged scattering from a silica sample measured with AGIPD and LAMBDA. The dashed line represents the form-factor fitting result, taking the polydispersity (Δ*R*/*R*) into account.

Fig. 3 ▸(*a*) bottom right shows a speckle pattern of silica particles obtained from a single bunch of PETRA III. Single (green pixels) and double (yellow pixels) photon events are clearly visible in the *Q* region < 0.04 nm^−1^. The average photon density in the SP mode was 0.014 photons pixel^−1^. The particle size of the sample was confirmed from the X-ray scattering profiles. Fig. 3 ▸(*b*) shows azimuthally averaged scattering from the summed speckle patterns acquired with both AGIPD and LAMBDA. The intensity profile of LAMBDA (Pennicard *et al.*, 2012 ▸) measured with a total exposure time of 100 s agreed with the calculated single-sphere form factor of *R* = 176 nm. Integrated intensity profiles collected with AGIPD in the SP and MP modes after gain correction (see Section S3) show that data are in very good agreement with results measured with LAMBDA. Thanks to the higher number of X-ray pulses in the MP mode, the photon statistics allow us to observe the form-factor oscillations at higher *Q* values.

**Figure 4:**
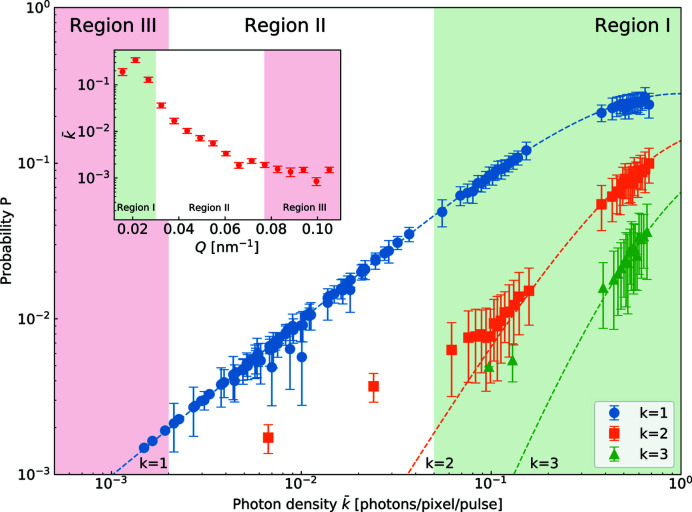
The observed probability of *k* = 1, 2 and 3 photons within a single pixel in SP mode as a function of mean photon density (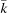). The dashed lines indicate the negative binomial distribution for β = 0.656. Photon density is divided into three regions according to the applied calculation methods for *g* ^(2)^. The inset shows 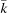 as a function of *Q* with corresponding regions.

Fig. 4 ▸ shows the probability of detecting *k* = 1, 2 and 3 photons in a pixel as a function of mean photon density (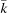) in the SP mode. The expected probabilities of the photon distribution for a speckle pattern can be described by a negative binomial distribution function expressed as (Goodman, 2010 ▸) 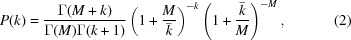
where Γ() represents the gamma function, and *M* is the number of speckle modes and is related to contrast via β ≡ *M* ^−1^. The contrast β equal to 0.656 was obtained from the global fit of equation (2) to the data shown in Fig. 4 ▸. The obtained value is consistent with the expected value of 0.65 (see Section S1). We divided the photon distribution into three regions. For 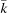 higher than 5 × 10^−2^ (region I), most of the experimental results follow the binomial distribution function very well. In the range 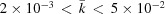 (region II), discrepancies between the data and expected values can be found, especially for a probability of detecting higher photon numbers (*i.e.* *k* = 2, 3). These disturbed photon statistics arise from incorrectly assigned photon numbers to a pixel caused by cross talk or charge sharing between neighboring pixels. In the very low count region of region III (*k* < 2 × 10^−3^), only single-photon events are present. The inset in Fig. 4 ▸ shows mean scattering intensity 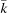 as a function of *Q* in the aforementioned regions.

We investigated the dynamics of the sample from the collected speckle patterns. For region I, we applied a conventional intensity correlation function *g* ^(2)^ defined as 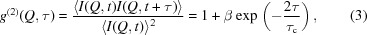 where β and τ are speckle contrast and delay time, respectively. *I*(*Q*, *t*) is the intensity at the wavevector *Q* and time *t*. The momentum transfer *Q* is defined by X-ray wavelength λ and the scattering angle θ according to *Q* = (4π/λ) sin (θ/2). The characteristic time of the investigated system is denoted as τ_c_ in equation (3) and is related to the free particle diffusion coefficient *D*_0_ = (τ_c_ *Q* ^2^)^−1^ for Brownian diffusion. The particle size *R* can be extracted from equation (S5) in the Supporting information. In region III where only single-photon events are detected, the event-correlator method was employed [equation (S8)].

**Figure 5:**
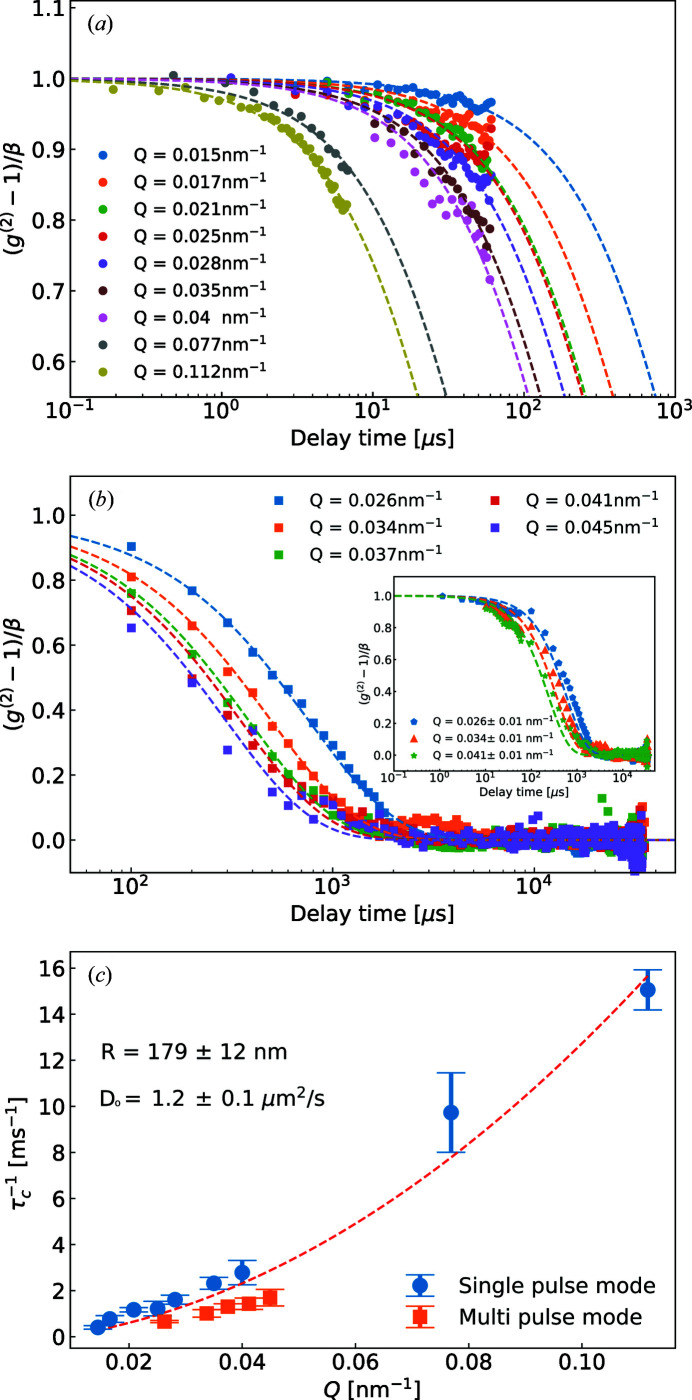
Normalized intensity autocorrelation functions in SP (*a*) and MP (*b*) mode. The normalized *g* ^(2)^ functions of experiments and corresponding fits of equation (3) to the data are shown by dotted, square and dashed lines in (*a*) and (*b*). The inset shows combined measured data from the SP and MP modes together with the corresponding *g* ^(2)^ functions calculated for particles with *R* = 179 nm. (*c*) The extracted τ_c_ from (*a*) and (*b*) as a function of *Q*. The dashed line shows a fitting curve of 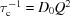.

We divided the detector plane into regions of interest (ROI) that are limited by contours of equivalent *Q* in which the average scattering varies by less than 10%, and calculated *g* ^(2)^ functions using equation (3). Figs. 5 ▸(*a*) and 5 ▸(*b*) show the normalized *g* ^(2)^ functions of different ROI. The dashed lines represent model fits of the right side of equation (3). In our measurements, the fastest correlation time τ_c_ was 66.4 µs at *Q* = 0.112 nm^−1^ with 192 ns temporal resolution. Both acquisition modes (*i.e.* SP mode and MP mode) were not able to capture individually the complete decay of the correlation curve. The correlation curves in the MP mode were normalized to the contrast value obtained in the SP mode. Fig. 5 ▸(*c*) shows the obtained τ_c_ from the fits as a function of wavevector transfer *Q*. The quality of the fits of the MP data is affected by the lack of early correlation points and initial contrast β. Consequently, the results show slightly longer relaxation times compared with the values obtained in the SP mode. The global fit to all τ_c_ values provides a *D*_0_ of 1.23 µm^2^ s^−1^ [see Fig. 5 ▸(*c*)]. Using the Stokes–Einstein relation [equation (S5)], we obtained *R* = 179 nm, which is in very good agreement with the value obtained from the form-factor fit [*R* = 176 nm, see Fig. 3 ▸(*b*)].

## 5. Discussion   

### 5.1. Signal-to-noise ratio (SNR)   

XPCS experiments are often performed at relatively large length scales (∼100 nm) and dynamics are probed at slow time scales (∼ms). This limitation is dictated mostly by the lack of sufficient coherent photon flux and the low-scattering cross section for X-rays. With the increase in coherent photon flux of diffraction-limited storage ring (DLSR) sources, the scope of XPCS can be extended to shorter time and length scales.

Based on the results of our studies and the predicted increase of intensity at PETRA IV, we calculate the expected measurement times for the XPCS studies according to (Möller *et al.*, 2019 ▸; Lumma *et al.*, 2000 ▸) 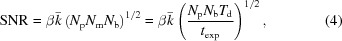
where *N*_p_, *N*_m_ and *N*_b_ represent the number of pixels, the number of frames in a burst and the number of bursts, respectively. *N*_m_ = *T*_d_/*t*_exp_, with *t*_exp_ being single-frame exposure time and *T*_d_ being total measurement time for *N*_m_.

**Figure 6:**
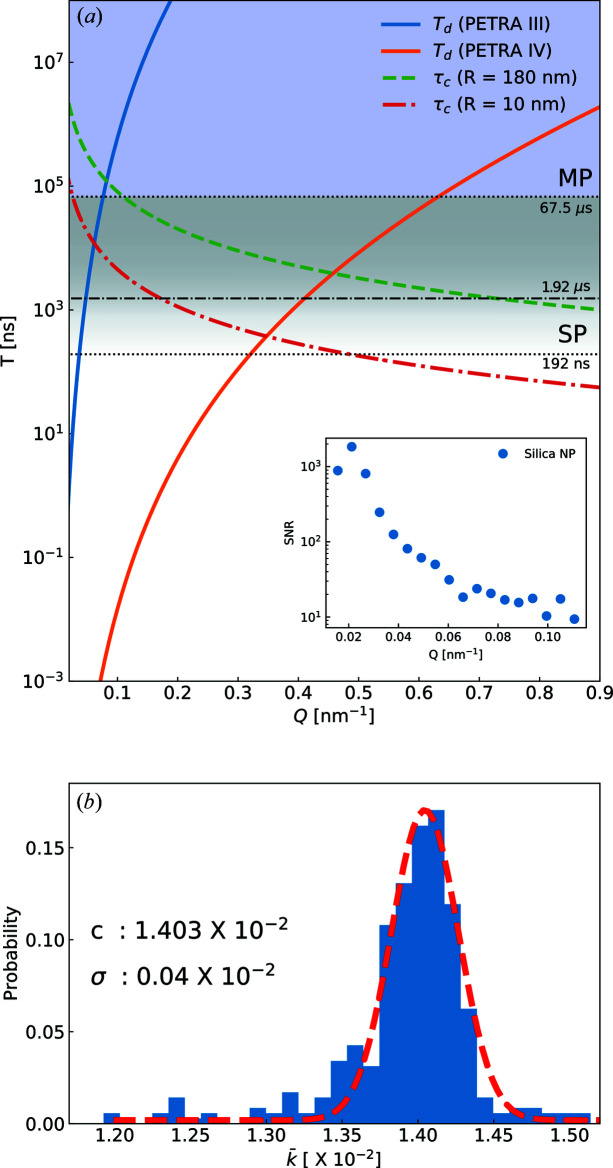
(*a*) Total experimental time expected for an XPCS experiment as a function of *Q*. The solid lines represent the required measurement time to achieve a SNR = 10 for PETRA III and PETRA IV. The expected characteristic time τ_c_ is shown for two different particle radii. The temporal range from 192 ns to 67.5 µs is accessed via SP mode. The horizontal dash–dotted line at 1.92 µs indicates expected achievable characteristic times. The range above 67.5 µs is covered by the MP mode. The SNR of our experiment is shown in the inset. (*b*) A histogram of the averaged total photons per single frame with a single Gaussian fit (red dashed line).

Fig. 6 ▸ shows the calculated total measurement time as a function of momentum transfer required for the XPCS studies based on the SNR of the current experiment (see the inset of the figure). The lowest limit of SNR = 10 is applied in the calculations. The solid lines are derived from equation (4). For the PETRA IV case, 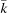 was multiplied with a factor of 400 and *N*_p_ = 10^6^ based on the higher brightness (Schroer *et al.*, 2018 ▸; Schroer, 2019 ▸) and the entire area of AGIPD, respectively. The areas above the solid lines represent the feasible region for XPCS studies. The result clearly implies that PETRA IV will allow us to investigate faster τ_c_ with a significant extension of the *Q* range to higher values. Additionally, the 256 times higher number of pixels compared with the current work also brings the advantage of measuring faster dynamics when the same experimental configurations are used. The dynamics of smaller particles at high wavevector transfers can be measured that are currently not accessible because of the low scattering intensity and too fast characteristic times.

However, the fastest temporal resolution is still limited by the X-ray bunch spacing and detector frame rates. AGIPD is able to acquire dynamics from 192 ns to 67.5 µs with the SP mode, and slower dynamics than 67.5 µs are accessible with the MP mode for both PETRA III and PETRA IV. Other fast 2D detector developments based on AGIPD technology (*e.g.* SPARTA) are ongoing and will provide up to 6.5 MHz frame rates. As a result, the advent of DLSR sources (*e.g.* PETRA IV) will make it possible to step up to investigate faster dynamics of low X-ray scattering systems, such as aqueous solutions of biological macromolecules (*e.g.* proteins). However, in order to access faster dynamics beyond the X-ray bunch spacing limit, split-pulse techniques will be required (Roseker *et al.*, 2009 ▸; Osaka *et al.*, 2016 ▸).

### 5.2. Stability   

One of the crucial factors for XPCS feasibility is the knowledge of pulse to pulse intensity fluctuation of the incoming X-ray because it influences β (Lee *et al.*, 2013 ▸; Lehmkühler *et al.*, 2018 ▸). The intensity fluctuation caused by the electron current of each bunch in the storage ring plays an important role in performing experiments. Since we acquired 352 single X-ray pulses delivered with a MHz repetition rate, the recorded data fluctuation becomes important, which includes not only the intensity fluctuation of X-rays but also detector responses. We assume that the mean intensity of a single frame is able to reflect the X-ray intensity of a single electron bunch, and the investigation of the histogram of 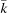 along the memory cell results in the σ value of 0.04 × 10^−2^ [see Fig. 6 ▸(*b*)].

## 6. Conclusions   

We have used the pulse nature of a synchrotron to demonstrate nanosecond X-ray photon correlation spectroscopy with 192 ns resolution using AGIPD. Correlation functions obtained from speckle patterns of freely diffusing silica particles in water show the expected microsecond dynamics in the investigated *Q* range (0.015–0.112 nm^−1^). The sample radius obtained from XPCS measurements shows very good agreement with the result obtained from the static small-angle scattering fit. This work provides an important step towards routine nanosecond XPCS experiments at fourth-generation DLSR sources.

## 7. Related literature   

The following references are only cited in the Supporting information for this article: Schätzel *et al.* (1988) ▸; Brown (1993) ▸. 

## Supplementary Material

Supporting information. DOI: 10.1107/S2052252520015778/it5023sup1.pdf
