# Supplementary material for: Nanosecond X-ray photon correlation spectroscopy using pulse time structure of a storage-ring source
Source: IUCrJ. 2021 Jan 1;8(Pt 1):124–30. doi: 10.1107/S2052252520015778 (PMC7792991; doi:10.1107/S2052252520015778)
Supplement: Supplementary file 1 [file m-08-00124-sup1.pdf]

IUCrJ

Volume 8 (2021)

Supporting information for article:

Nanosecond X-ray photon correlation spectroscopy using pulse time structure of a storage-ring source

Wonhyuk Jo, Fabian Westermeier, Rustam Rysov, Olaf Leupold, Florian Schulz, Steffen Tober, Verena Markmann, Michael Sprung, Allesandro Ricci, Torsten Laurus, Allahgholi Aschkan, Alexander Klyuev, Ulrich Trunk, Heinz Graafsma, Gerhard Grübel and Wojciech Roseker

Nanosecond X-ray Photon Correlation Spectroscopy using pulse time structure of storage ring source : Supporting information

S1. Contrast Calculation

We calculate the expected speckle contrast β as a function of beam size and scattering wave vector Q based on our experimental parameters. The total speckle contrast β_{total} can be expressed by (Möller *et al.*, 2019)

$$\beta_{\mathrm{total}}(d_{\mathrm{b}}, d_{\mathrm{t}}, Q, \lambda, L) = \beta_{\mathrm{cl}}(d_{\mathrm{b}}, d_{\mathrm{t}}, Q, \lambda)\beta_{\mathrm{res}}(d_{\mathrm{b}}, L, \lambda), \tag{S1}$$

where β_{cl} represents the effect of transverse and longitudinal coherence lengths. The β_{res} corresponds to the finite angular resolution of the experimental setup. $d_{\mathrm{b}}, d_{\mathrm{t}}, \lambda$, and L represent the beam size, sample thickness, X-ray wavelength, and sample to detector distance, respectively.

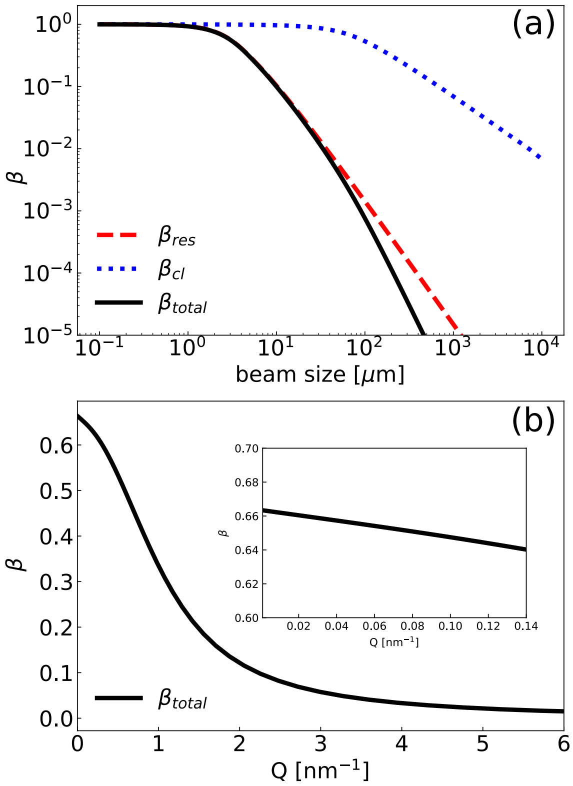

Fig. S1. Speckle contrast β as a function of beam size (a) and Q (b) based on our experimental conditions in Tab. S1 . For the SAXS regime that we performed current measurement, β shows about 0.65 in inset figure.

Also, β_{cl} and β_{res} are given by

$$\begin{aligned}&\beta_{\mathrm{cl}}(d_{\mathrm{b}}, d_{\mathrm{t}}, Q, \lambda)\\&= \frac{2}{(d_{\mathrm{b}} \times d_{\mathrm{t}})^2} \int_0^{d_{\mathrm{b}}} dx \int_0^{d_{\mathrm{t}}} dz (d_{\mathrm{b}} - x)(d_{\mathrm{t}} - z) \exp(-x^2/\xi_{\mathrm{h}}^2)\\&\times [\exp(-2|Ax + Bz|) + \exp(-2|Ax - Bz|)],\end{aligned} \tag{S2}$$

$$\beta_{\mathrm{res}}(d_{\mathrm{b}}, L, \lambda) = \left\{ \frac{2}{w^2} \int_0^w (w - v) \left[\frac{sin(v/2)}{v/2} \right]^2 dv \right\}^2, \tag{S3}$$

where $w = 2\pi d_{\mathrm{p}} d_{\mathrm{b}}/L\lambda$. d_{p} and ξ_{h} are detector pixel size, and horizontal coherent length respectively. The other parameters are defined with $A = (\Delta\lambda/\lambda)Q\{1 - [(1/4)Q^2/k^2]\}^{1/2}$, $B = -(\Delta\lambda/2\lambda)(Q^2/k)$, and $k = 2\pi/\lambda$. The result of theoretical

calculated value of β is shown in Fig. S1 based on our experimental configurations (Tab. S1).

Table S1. *Experimental parameters of current work.*

λ [nm]	L [m]	d_p [μm]	d_b [μm]	d_t [μm]	ξ_h [μm]	$\Delta\lambda/\lambda$
0.155	5	200	2.5	700	50	1.4e-4

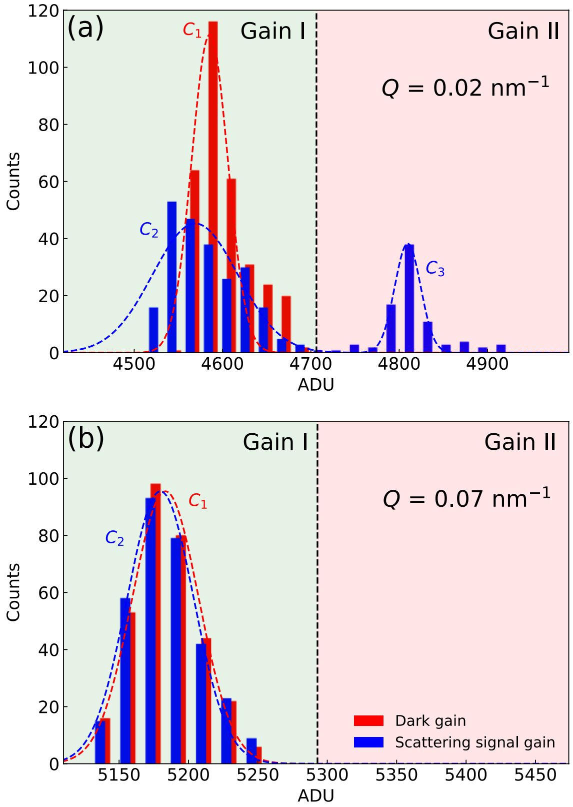

Fig. S2. Histograms of gain values of the scattering data and darks from two different selected single pixel measured at (a) low scattering vector $Q = 0.02$ nm^{-1} and (b) high scattering vector $Q = 0.07$ nm^{-1}. Fit parameters c_1, c_2, and c_3 denote the center of each Gaussian fit to the histograms. Vertical dashed lines separating the Gain I and Gain II region represent the threshold levels defined by the width of Gaussian fits for the gain switching.

S2. Gain switching for MP mode

We used the gain of dark data to define the gain switching based on histograming of scattering data and dark gains. Fig. S2 shows histograms from the scattering data and

dark frame gain values for arbitrary chosen single-pixels according to the incoming photon intensities. In Fig. S2(a), we can clearly distinguish two peaks in the gain profile of the scattering data, but only a single peak is visible in the histogram of the dark gain. Fit parameters c_1, c_2, and c_3 represent the centers of each Gaussian fitted to the aforementioned histograms. Note that, c_2 is close enough to c_1, which indicates the equivalent gain states. Hence, the dark mode gain states can be assigned to Gain I, which is the default value, the elements of the Gaussian peak c_3 can be regarded as switched gain states (i.e., Gain II). At higher Q values, both data sets (scattering signal and dark frames) show similar histograms, as shown in Fig. S2(b). This indicates no gain switching at higher Q values (Q = 0.07 nm^{-1}) due to the low scattering intensity. By applying the aforementioned method, we extracted gain states for every single pixel and memory cell. This procedure was especially essential for the low Q region where the intensity was high enough to switch the gain of pixels. The difference between c_2 and c_3 in Fig. S2(a) show a deviation from the work performed by (Mezza *et al.*, 2019) due to the different operation mode.

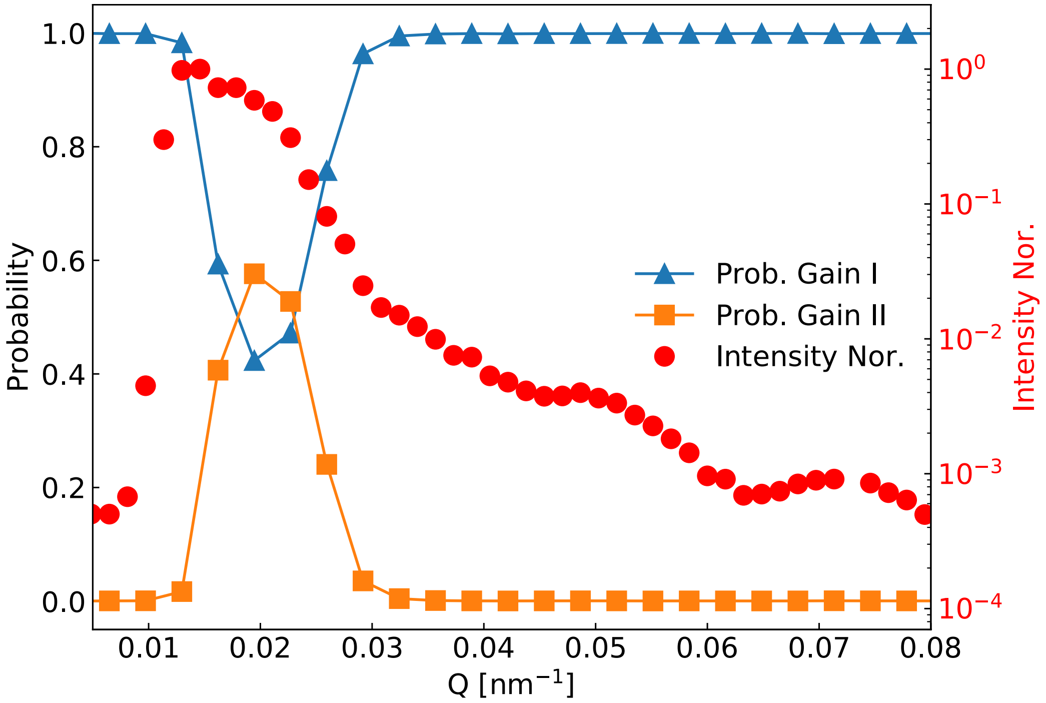

Fig. S3. The probabilities for gain states. The high intensity regime shows high probability for Gain II.

AGIPD is capable of switching the gain state depending on the incoming photon intensity with MHz speed. Here we show that the gain switching took place only in the low Q regime by calculating the probabilities of gain states for the MP mode in Fig. S3. According to this result, the probability of Gain II becomes negligible, but the probability of Gain I becomes dominant from $Q = 0.03$ nm^{-1} where the intensity is about 100 times less than the maximum intensity ($Q = 0.02$ nm^{-1}). Therefore, for the SP mode, we are able to assume that all gain states remain with the default value (Gain I) because the number of accumulated X-ray pulses per single frame is 520 times less than the MP mode.

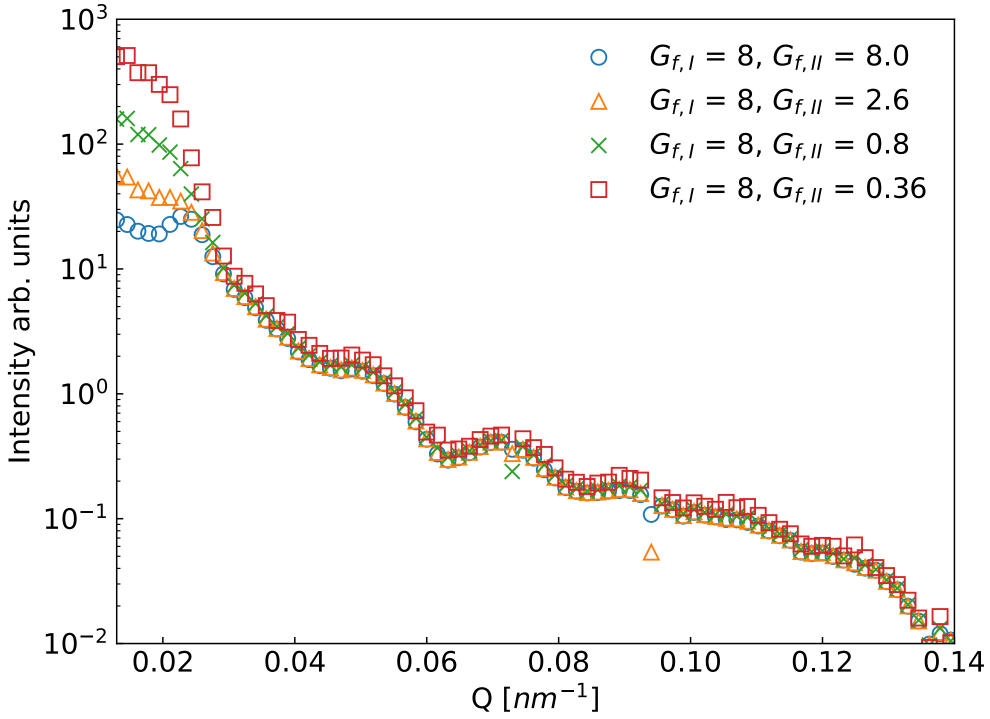

Fig. S4. Azimuthally averaged intentity profiles as function of Q with various $G_{\mathrm{f,II}}$s. We found $G_{\mathrm{f,II}} = 0.36$ gives the most reliable intensity profiles.

S3. Gain factors in MP mode

We defined the gain factors (G_{f}) to convert intensities from signal value with

$$I = \frac{Signal(ADU)}{G_{\mathrm{f}} \times E_{\text{X-ray}}}, \tag{S4}$$

where $E_{\text{X-ray}}$ is X-ray photon energy (8 keV). The factor of Gain I ($G_{\mathrm{f,I}}$) can be deduced to 8 ADU/keV by defining a photon converting factor (64 /ADU) at the single photon region in Fig. 2.

Based on the $G_{\mathrm{f,I}} = 8$, we can estimate the $G_{\mathrm{f,II}}$ by applying to the azimuthally averaged intensity profiles in Fig. S4. Since the most of gain switched pixels are located

at low Q region, where the scattered intensities are strong, the variation of $G_{\mathrm{f,II}}$ mainly changes low Q intensity profiles. In our experiment, we chose $G_{\mathrm{f,II}} = 0.36$ which is 25 times higher gain values than $G_{\mathrm{f,I}} = 8$ after the intensity conversion.

S4. Analog-to-digital units in SP mode

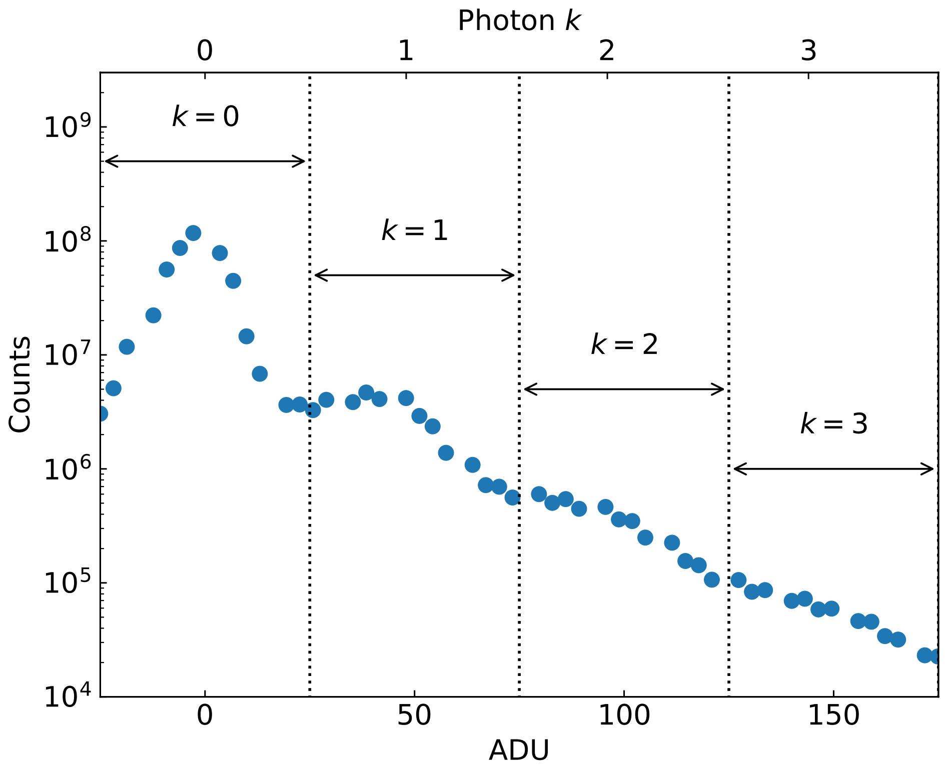

Fig. S5. Histogram of analog-to-digital units obtained from the SP mode. The vertical dashed lines show selected ranges for the conversion of ADUs into an integer number of photons.

Fig. S5 shows an analog-to-digital units histogram of the SP mode data. The first peak of the histogram is centered at 50 ADUs and corresponds to the first photon peak. The noise of the detector was derived from the σ value (5 ADUs) of the zero photon peak. Lower value of the ADU in the SP mode compared to the MP mode is

due to different operation mode of the detector.

S5. XPCS data analysis

S5.1. Intensity autocorrelation function

Photon correlation spectroscopy with a two-dimensional detector was first developed for dynamic light scattering with visible laser (Dorfmüller, 1992; Cipelletti & Weitz, 1999) and extended to X-rays (Dierker *et al.*, 1995*b*; Lumma *et al.*, 2000). In the speckle analysis, the intensity correlation functions $g^{(2)}(\tau)$ can be calculated by equation (3). It can be expressed with contrast β and $g^{(1)}$ which are related to an exponential decay function via the Siegert relation (see the main text equation (3)). Since τ_c is related to the diffusion coefficient $D_0 = (\tau_c Q^2)^{-1}$ for typical Brownian diffusion, we could extract the particle size R according to the Stokes-Einstein relation.

$$R = \frac{k_b T}{6\pi\eta D_0}, \tag{S5}$$

where k_b, T and η are Boltzmann constant, temperature and fluid viscosity, respectively. Furthermore, we applied a symmetric normalization function for more accurate $g^{(2)}$ calculation, which is taking the time evolution of experimental condition into account. The denominator of equation (3), $\langle I(Q,t)\rangle^2$, offers the standards normalization for point correlator experiment (Brown W, 1993). It represents that all speckle measured during the experiment would be averaged out and does not reflect long-term and long-range variations of the illumination source. The simple normalization equation has been acceptable for a photon correlation spectroscopy using a visible laser source offering stable intensity variation. On the other hand, most of the coherent X-ray sources have not even shot to shot intensity fluctuation but also long-term intensity changes compared with the scan duration. Thus, to avoid the long-term intensity drift of illumination source, the symmetric normalization scheme was developed (Schätzel

et al., 1988) with

$$g^{(2)}(Q,\tau) = \frac{\langle I(Q,t)I(Q,t+\tau)\rangle_Q}{\langle I(Q,t)\rangle_{Q,t_{\mathrm{left}}}\langle I(Q,t)\rangle_{Q,t_{\mathrm{right}}}} \tag{S6}$$

where $\langle I(Q,t)\rangle_{q,t_{\mathrm{left,right}}}$ is able to be shown,

$$\langle I(Q,t)\rangle_{Q,t_{\mathrm{left}}} = \frac{1}{N_\tau}\sum_{i=1}^{N_\tau} I(Q,i), \tag{S7a}$$

$$\langle I(Q,t)\rangle_{Q,t_{\mathrm{right}}} = \frac{1}{N_T - N_\tau}\sum_{i=N_\tau}^{N_T} I(Q,i), \tag{S7b}$$

where τ, N_τ and N_T represent delay time, index frame of the delay time, and index of the total frame respectively.

S5.2. Event correlation

The event correlation was applied for analyzing XPCS to minimize computer requirements compared to the standard intensity correlation method (Chushkin *et al.*, 2012). Since this method is based on counting the number of pixels where photons are detected, it has the advantage to not only simplify the analyzing process but also to reduce the analysis time.

Most importantly, the event correlation does not consider multi-photon events, which does not mean a resolving of more than one-photon events. This method, thus, is suitable for the low scattering intensity regime, where the zero or one photon events are usually dominant. The principle of an event correlation is to build the correlation function from photon events detected in a pixel p at time t represented by the list $e(t,p)$ as

$$g^{(2)}(\tau) = \frac{\left\langle \sum_p e'(t+\tau,p)\right\rangle_t}{\langle \overline{e(t,p)}^2\rangle_t}, \tag{S8}$$

where

$$e'(t+\tau,p) = \begin{cases} 1 & \text{if} \quad p \in S(t,\tau) \\ 0 & \text{otherwise.} \end{cases} \tag{S9}$$

$S(t,\tau)$ is the set of pixels which was exposed by photons at both time t and time $t+\tau$, and $\overline{e(t,p)}$ denotes the total number of photons registered by the entire AGIPD at time t normalized by the total number of pixels.

Most of the pixels show a zero event from extremely weak scattering intensities of X-ray single-shot irradiation, especially at the higher Q regime. Consequently, the zero count pixels are omitted in this calculation, and it will help to significantly speed up data processing speed. Accordingly, this shows accurate results only for low photon intensity region, where the one photon events are dominant.
